# *In vitro* characterization of novel hyaluronan-antioxidant conjugates as potential topical therapeutics against hearing loss

**DOI:** 10.3389/fphar.2024.1355279

**Published:** 2024-02-28

**Authors:** Elizabeth M. Arrigali, Joachim G. S. Veit, Bhaskar Birru, Jack Van Tine, Kolton C. Sandau, Emma Barrett-Catton, Zachary Tonnerre, Monica A. Serban

**Affiliations:** ^1^ Department of Biomedical and Pharmaceutical Sciences, University of Montana, Missoula, MT, United States; ^2^ Montana Biotechnology Center (BIOTECH), University of Montana, Missoula, MT, United States

**Keywords:** oxidative stress, hyaluronan, hearing loss, ototherapeutic, antioxidant

## Abstract

Noise-induced hearing loss affects roughly 430 million people worldwide. Current treatment options often require invasive medical procedures, and to date, there are no FDA-approved drug therapies. While the causes can be diverse, noise induced hearing loss is unequivocally associated with oxidative stress and inflammation, and subsequent damage to the inner ear structures. Several studies have shown that various antioxidants such as glutathione, cysteine, and methionine can be used to mitigate oxidative damage from reactive oxygen species; however, these studies relied on invasive or systemic drug delivery methods. This study focused on the development and characterization of a novel series of antioxidant compounds that would be suitable for non or minimally invasive topical inner ear delivery and could mitigate reactive oxygen species associated cellular damage. Specifically, a series of covalent conjugates were synthesized by using hyaluronan as a drug carrier, and methionine, cysteine or glutathione as antioxidant drugs. The conjugates were tested for their ability to readily permeate though *in vitro* round window membrane and tympanic membrane permeation models, as well as their *in vitro* internalization into cochlear cells. Our data revealed interdependence between the molecular weight of the hyaluronan carrier, and the tissue and cellular membrane permeation capacity. Subsequent screening of the adequately sized conjugates in* in vitro* acellular assays revealed the strongest antioxidant activity for the cysteine and glutathione conjugates. These oxidative stress protective effects were further confirmed in cellular *in vitro* assays. Collectively, the data herein showcase the potential value of these conjugates as therapeutics against oxidative-stress-mediated cellular damage specific to noise-induced hearing loss.

## 1 Introduction

Hearing loss affects over 5% of the global population or roughly 430 million people (WHO, 2023). Some causative agents include drugs like chemotherapeutics or aminoglycosides, diseases such as viral or bacterial infections, natural aging processes, loud noises, and repeated ear infections (Mayo Clinic, 2023; WHO, 2023). Based on the ear structures affected, hearing loss can be categorized into three types: conductive, sensorineural (SNHL), or mixed. SNHL is the most common type of hearing loss and involves permanent damage to inner ear structures including the organ of Corti, auditory nerve, or central nervous system (Alexander and Harris, 2013; Tanna et al., 2023). Regardless of the causative agents, the mechanisms of SNHL invariably involve the production of reactive oxygen species (ROS), inflammation, glutamate excitotoxicity, calcium overload and disturbances to energy metabolism (Mao and Chen, 2021). Therefore, many studies focused on developing therapeutics or prophylactics against SNHL target the reduction of ROS and/or inflammation (Butler and van Der Voort, 2000; Hoshino et al., 2010; Clifford et al., 2011; Mukherjea et al., 2015; Ma et al., 2019; McCrary et al., 2019; Nyberg et al., 2019; Pak et al., 2020a; Pecha et al., 2020; Mao and Chen, 2021). Nevertheless, there is still a substantial need for FDA-approved prophylactics or therapeutics against hearing loss (Stern et al., 2005; Nyberg et al., 2019; Health, 2023).

Previous animal studies have highlighted the potential value of using antioxidants to mitigate SNHL (Le Prell et al., 2007; Cheng et al., 2008; Tavanai and Mohammadkhani, 2017; Pecha et al., 2020). In these studies, the primary challenges were reaching a high enough therapeutic concentration of antioxidant within the cochlea (Nyberg et al., 2019; Steyger, 2021). In order to reach the cochlea via systemic delivery, a therapeutic would need to cross the blood-labyrinth barrier, which similarly to the blood-brain barrier, poses significant hurdles to adequate drug availability. Conversely, topical drug delivery would require drug passage across the tympanic membrane (TM), a thin tissue that separates the outer ear and middle ear, and the round window membrane (RWM), which separates the middle ear and inner ear (Ma et al., 2019; Nyberg et al., 2019; Mao and Chen, 2021; Steyger, 2021). The mechanism of ROS production in noise-induced hearing loss (NIHL), a type of SNHL, has been previously investigated (Dinh et al., 2015a; Kurabi et al., 2017a; Fetoni et al., 2019; Varela-Nieto et al., 2020; Fetoni et al., 2022). Acoustic trauma leads to mechanical damage, such as rupture and/or displacement of the TM, displacement of the basilar membrane, shearing of the stereocilia of hair cells, and injury to the supporting cochlear cells (Nicotera et al., 2003; Dinh et al., 2015a; Raju, 2015). In addition to mechanical damage, pro-inflammatory cytokines are released which initiate additional inflammatory and cellular death pathways, as well as ROS generation (Kamata et al., 2005; Jiang et al., 2006; Dinh et al., 2015a; Kurabi et al., 2017a; Fetoni et al., 2019; Frye et al., 2019; Wu et al., 2020; Fetoni et al., 2022). This subsequently leads to permanent damage to cochlear structures including the organ of Corti (Kurabi et al., 2017a; Frye et al., 2019; Wu et al., 2020). Repetitive acoustic trauma, was also shown to lead to swelling of the stria vascularis, resulting in additional generation of ROS. Although animal studies have explored the successful use of antioxidants to target ROS associated with NIHL, and the mechanism of ROS generation is understood, human data lag (Dinh et al., 2015a; Pak et al., 2020a; Abbasi et al., 2021). As previously mentioned, adequate drug delivery of prophylactics or treatments to the cochlea where the ROS production occurs, is challenging. Our lab has previously developed and characterized a topical hyaluronan (HA)—D-methionine (M) covalent conjugate (M-HA) (Arrigali and Serban, 2022), which was shown to be effective against cellular ROS damage and readily permeated an *in vitro* RWM permeation model.

When used as an additive, HA, a polymeric glycosaminoglycan endogenous to all mammalian systems, has been previously reported to aid in the delivery of drugs to the cochlea (Shibata et al., 2012; Rogha and Kalkoo, 2017). Structurally, HA is comprised of repeating disaccharide units consisting of a β-1,4-D-glucuronic acid and a β-1,3-N-acetylglucosamine (Gupta et al., 2019), lending itself to facile chemical modifications that typically yield highly biocompatible derivatives (Serban and Skardal, 2019). This study expands on our previous work, explores additional HA-antioxidant conjugates using cysteine and glutathione as antioxidant components, and reveals important conjugate synthesis considerations that translate to therapeutic efficiency. Specifically, we show that the molecular weight (MW) of HA affects its ability to effectively permeate previously-developed *in vitro* permeation models of the TM and RWM (Veit et al., 2022a; Veit et al., 2022b; Singh et al., 2022), as well as its cellular internalization into cochlear cells. Moreover, we show that the HA-antioxidant conjugates have antioxidant properties both in *in vitro* acellular and cellular assays, and they can permeate *in vitro* models of the RWM and TM. Together, these results show the HA-antioxidant conjugates developed in this study may have potential as topical therapeutics against NIHL.

## 2 Materials and methods

### 2.1 Materials

The following reagents and consumables have been used for this study: HA of five molecular weights (31 kDa, 146 kDa, 285 kDa, 608 kDa, and 1,030 kDa (Lifecore Biomedical Chaska, MN)), bis-tris buffer pH 6.0 (Thermo Scientific, Waltham, MA), 1-ethyl-3-(3-dimethylaminopropyl)carbodiimide hydrochloride (EDC, Thermo Scientific, Waltham, MA), sodium chloride (NaCl, Thermo Scientific, Waltham, MA), iodoacetic acid (Thermo Scientific, Waltham, MA), ethanol absolute (EtOH, VWR,Radnor, PA), Dulbecco’s phosphate buffered-saline (DPBS, Corning, Corning, NY), #2 Whatman paper (Thermo Scientific, Waltham, MA), deuterated water (D_2_O, Thermo Scientific, Waltham,MA), 70 mL 3,500 kDa MW cut-off (MWCO) dialysis cassettes (Thermo Scientific, Waltham, MA), HA-BODIPY of two molecular weights (24 kDa and 240 kDa) (Echelon Biosciences, Salt Lake City, UT), D-methionine (Thermo Scientific, Waltham, MA), cysteine (Thermo Scientific, Waltham, MA), glutathione-reduced (Thermo Scientific, Waltham, MA), glutathione-oxidized (Thermo Scientific, Waltham, MA), N-hydroxysuccinimide (NHS, Thermo Scientific, Waltham, MA), dithiothreitol (DTT, Thermo Scientific, Waltham, MA), 12 N hydrochloric acid (HCL, Thermo Scientific, Waltham, MA), sodium hydroxide (NaOH, Thermo Scientific, Waltham, MA), potassium phosphate buffer (Thermo Scientific, Waltham, MA), boric acid (Thermo Scientific, Waltham, MA), potassium hydroxide (Thermo Scientific, Waltham, MA), mercaptoacetic acid (Thermo Scientific, Waltham, MA), o-phthalaldehyde (OPA, Thermo Scientific, Waltham, MA), isopropanol (IPA, VWR, Radnor, PA), Gemini 3 µm C18 110Å column (Phenomenex, Torrance, CA), nitro blue tetrazolium chloride (NBT, Thermo Scientific, Waltham, MA), phenazine methosulfate (PMS, Thermo Scientific, Waltham, MA), β-nicotinamide adenine dinucleotide reduced disodium salt (NADH, Thermo Scientific, Waltham, MA), L (+)- ascorbic acid (Thermo Scientific, Waltham, MA), hydrogen peroxide (H_2_O_2_, Millipore Sigma, St. Louis, MO), Superoxide anion assay kit (Millipore Sigma, St. Louis, MO), UV-transparent microplates (Corning, Corning, NY), Fisherbrand 96-well white opaque plates (12566619, Thermo Scientific, Waltham, MA), Corning Spin-X centrifuge tube filter (0.22 µm cellulose acetate, Corning, Corning, NY), measure-IT thiol assay kit (Thermo Scientific, Waltham, MA), PL Aquagel OH mixed-H SEC column (Agilent, Santa Clara, CA), micro bicinchonic acid protein assay kit (BCA, Thermo Scientific, Waltham, MA), (3-4,5-dimethyl thiazole 2-yl) 2,5-diphenyltetrazoliumbromide (MTT, Thermo Scientific, Waltham, MA), and Pierce immobilized TCEP disulfide reducing gel (Thermo Scientific, Waltham, MA).

The following cell lines and cell culture reagents have been used for this study: House Ear Institute-Organ of Corti (HEI-OC1, Kalinec lab, UCLA, Los Angeles, CA), fetal bovine serum (FBS, Corning, Corning, NY), Dulbecco’s modified Eagle medium (DMEM, Corning, Corning, NY), CellTiter 96 AQueous One Solution cell proliferation assay (MTS assay, Promega, Madison, WI), CyQUANT lactate dehydrogenase (LDH) cytotoxicity assay (Invitrogen, Waltham, MA), chloromethyl derivative of 2′,7′-dichlorodihydrofluorescein diacetate (CM-H_2_DCFDA, Thermo Scientific, Waltham, MA), hydrogen peroxide (H_2_O_2_, Millipore Sigma, St. Louis, MO), trypsin-ethylenediaminetetraacetic acid (trypsin-EDTA, Corning, Corning, NY), human primary neonatal epidermal keratinocytes (Gibco, Burlington, MA), Epilife media (Gibco, Burlington, MA), human keratinocyte growth supplement (HKGS, Gibco, Burlington, MA), calcium chloride (Thermo Scientific, Waltham, MA), 2-phospho-L-ascorbic acid trisodium salt (Sigma, St. Louis, MO), human keratinocyte growth factor (hKGF, Sigma, St. Louis, MO), primary small airway epithelial cells (ATCC, Manassas, VA), airway epithelial cell basal medium (ATCC, Manassas, VA), bronchial epithelial cell growth kit (ATCC, Manassas, VA), Pneumacult-ALI medium (Stemcell, Vancouver, Canada), and Hank’s balanced salt solution (HBSS, Thermo Scientific, Waltham, MA).

The following instruments have been used for this study: Bruker 400 with BBO broadband probe and 60 sample auto express autosampler (Bruker, Billerica, MA), Agilent Biotek Cytation 5 imaging multi-mode microplate reader (Santa Clara, CA), Agilent 1,260 Infinity II HPLC instrument with a UV-VIS detector (Agilent, Santa Clara, CA) equipped with an OptiLab differential refractive index (RI) detector (Wyatt Technologies, Santa Barbara, CA), and a miniDawn 3-angle/multi-angle light scattering (MALS) detector (Wyatt Technologies, Santa Barbara, CA), and Malvern Zetasizer Ultra advanced light scattering system with folded capillary zeta cells (Malvern Panalytical, Westborough, MA).

### 2.2 Synthesis of various molecular weight carboxymethylated HA (CMHA)

CMHA was synthesized as previously described (Arrigali and Serban, 2022), using five different molecular weights of HA (31 kDa, 146 kDa, 285 kDa, 608 kDa, and 1,030 kDa). Briefly, hyaluronan (1,000 mg) was added to 45% W/V NaOH (10 mL) and allowed to activate for 2 h. Prior to the end of the 2 h, iodoacetic acid (1.98 g) was dissolved in IPA (25 mL). The iodoacetic acid solution was added to the HA/NaOH solution and then IPA (75 mL) was added. The reaction mixture was stirred for 2 h. After reacting, the solution was filtered with filter paper and the precipitate was solubilized in water (100 mL) and neutralized to a pH of 7.0. The reaction solution was then loaded into a dialysis cassette and dialyzed against H_2_O with 3 changes per 24 h for a total of 72 h. The percent of carboxymethylation was determined using the previously established efficiency protocol via ^1^H-NMR (Arrigali and Serban, 2022).

### 2.3 Synthesis of HA-antioxidant conjugates (M-HA, C-HA, and G-HA)

M-HA was synthesized following a previously established protocol (Arrigali and Serban, 2022) using five different MW HA starting materials for CMHA (31 kDa, 146 kDa, 285 kDa, 608 kDa, and 1,030 kDa). The stoichiometry of the reaction was 8:1 mol of methionine to moles of CMHA carboxy, and 3:1 mol of EDC to moles of CMHA carboxy. Briefly, CMHA (200 mg) was dissolved in MES buffer (40 mL) and D-met (880 mg) and EDC (400 mg) were then added to the reaction mixture and allowed to react for 24 h. After 24 h, the reaction solution was neutralized and loaded into a dialysis cassette and dialyzed against H_2_O for 72 h with 3 water changes a day.

Synthesis of C-HA was performed based on a previously published protocol (Arrigali and Serban, 2022), but with modified reaction stoichiometry and addition of NHS for stabilization. A 1.65:1 ratio of moles of cysteine to moles of CMHA carboxy, 6.5:1 mol of EDC to moles of CMHA carboxy, and 3:1 mol of EDC to moles of NHS was used for this reaction. Preliminary reactions consisted of 100 mg of CMHA, which was scaled up to 500 mg of CMHA in subsequent batches. Briefly, 100 mg of CMHA (31 kDa HA starting material) was solubilized in 20 mL of water and the pH was adjusted to 5.5. The reaction mix was stirred until fully solubilized (∼5 min). EDC (466 mg) and NHS (92 mg) were added to the CMHA solution and stirred vigorously for 15 min. Cysteine (100 mg) was solubilized in water (2 mL) and then it was added to the CMHA/EDC/NHS mixture. The reaction stirred for 6 h at RT. After 6 h, the solution was placed in dialysis cassettes with a MWCO of 3,500 and dialyzed against water with 1% w/v NaCl and 0.2 mM HCl for 48 h, with 3 water changes per day. After 48 h the solution was dialyzed against water with 0.2 mM HCl for another 48 h with 3 changes per day. Subsequently, the solution was dialyzed against water for 1 h before removing from dialysis cassettes, transferring to a suitable container, and placing in freezer prior to lyophilization. The reaction yielded 94.53 mg of C-HA. ^1^H-NMR was performed as described below.

G-HA synthesis was conducted as described for C-HA, substituting the molar equivalency of glutathione-oxidized and glutathione-reduced in place of cysteine. For the glutathione-oxidized reaction product, the intrinsic disulfide bonds were reduced with DTT. DTT (92.5 mg) was added to the reaction solution and the pH was adjusted to 8.5 with NaOH. The reaction was stirred overnight at RT. The next morning, the pH was decreased to 3.5 with HCl, and the solution was then dialyzed as described for C-HA. GO-HA reaction yielded 80.2 mg and GR-HA reaction yielded 119.99 mg. ^1^H-NMR was performed for both reaction products as outlined below.

### 2.4 ^1^H-NMR analysis of compounds

To confirm the structures and purity of CMHA and conjugates, ^1^H-NMR spectral data were obtained using a Bruker 400 at 20°C. For the analyses, samples were dissolved in deuterium oxide at a concentration of 5 mg/mL and all spectra were referenced to the residual solvent peak (D_2_O) at δ = 4.65 ppm.

### 2.5 Determination of conjugation efficiency

The methionine content for each batch of M-HA was determined according to a previously published protocol (Veit et al., 2023).

The quantity of thiol containing antioxidant in C-HA or G-HA was determined using the measure-IT Thiol Assay kit. Two (2) volumes (relative to volume of sample to be added) of immobilized TCEP disulfide reducing resin were added to a spin-X centrifuge tube filter (0.22 µm cellulose acetate) and centrifuged (2 min, 100*rcf) to remove liquid. The filters were transferred to fresh collection tubes and one volume of samples/standards was added to the reducing resin. These were placed onto a shaker for 75 min at room temperature to allow complete sample disulfide reduction. Samples were then centrifuged (2 min, 100*rcf) and the flow-through containing the reduced sample was collected. Thiol quantification was then determined using a measure-IT Thiol Assay Kit per manufacturers protocol. Briefly, 100 μL of kit quantification buffer was added to the well of a 96 well plate. Sample (10 μL) was then dispensed into the well and mixed via pipetting. After 30 min sample fluorescence was measured by plate reader (excitation/emission: 494/517 nm).

### 2.6 Molecular weight of conjugates

The molecular weight and polydispersity of CMHA and CMHA-antioxidant conjugates were determined by size exclusion chromatography with multi-angle light scattering as described in a previously established protocol (Veit et al., 2023).

### 2.7 Zeta-potential of conjugates

The zeta-potentials of the conjugates C-HA, G-HA, and M-HA were determined using the Zetasizer Ultra. A 5 mg/mL solution was loaded into cuvettes using between 700 and 800 µL for each run.

### 2.8 Cellular internalization of HA-BODIPY

Mouse cochlear HEI-OC1 cells were seeded in 6-well plates and allowed to proliferate until confluent. The cells were then treated with 24 kDa or 240 kDa HA-BODIPY at 250 μg/mL in 0.9 mL growth media for the indicated amount of time. Once treated, the cells were rinsed with 1.5 mL chilled DPBS, then released with 0.05% trypsin/EDTA. The cells were then transferred to a 1.5 mL microfuge tube, centrifuged for 5 min (500*rcf, 4°C) and the pellet was washed three times with 1.5 mL chilled DPBS. The pellet was then resuspended in 0.25 mL nanopure water and mechanically lysed over three repetitions of the following: Freeze cells at −80°C; thaw then centrifuge the cells for 15 min (16,000*rcf, 4°C); use sonicating water bath to disrupt and homogenize pellet, briefly vortex; repeat. Once lysed, the solution was stored at −20°C until use. Fluorescence was read on a plate reader (excitation/emission: 485/530 nm) and HA-BODIPY content was determined against a standard curve of each HA-BODIPY. A BCA assay was used to determine lysed protein concentration and normalize the results to total cell lysate protein.

### 2.9 Permeation and cytocompatibility of HA-conjugates in TM and RWM models

*In vitro* RWM and TM 3D tissue permeation models were cultured and compound permeation was evaluated as previously described (Veit et al., 2022a; Singh et al., 2022). Briefly, the TM models were composed of human primary neonatal epidermal keratinocytes proliferated with Epilife complete media [Epilife with HKGS and penicillin/streptomycin (0.5%)]. The cells were seeded into 12 mm cell culture inserts (1.5 × 10^5^ cells/insert) in a 6-well plate with Epilife complete media supplemented with calcium chloride (1.44 mM) and incubated overnight (37°C and 5% CO_2_). The following day the media was aspirated and replaced with Epilife complete media (1.5 mL) supplemented with calcium chloride (1.44 mM), 2-phospho-L-ascorbic acid trisodium salt (91.4 μg·mL^−1^), and hKGF (10 ng·mL^−1^). This media was changed every 2 days, and an air-liquid interface (ALI) was created by drying the surface of the tissue for the remainder of the tissue growth. The RWM models were composed of primary human small airway epithelial cells cultured in epithelial cell basal media supplemented a with bronchial epithelial growth kit. During the ALI growth periods, PneumaCult™-ALI complete media (PALI) was used (PneumaCult™-ALI Basal Medium supplemented with PneumaCult™-ALI Maintenance Supplement, PneumaCult 10x Supplement, 4 μg/mL Heparin Solution, and 0.48 μg/mL hydrocortisone). The cells were seeded in 12 mm cell culture inserts (1 × 10^5^ cells/insert) in 12-well plates with complete small airway growth media. Media changed after 24 h in both the basal and apical chambers. After 72 h, the basal media was replaced with PALI and the ALI was established. The tissues were grown at ALI for 14 days with media changes every 2 days. For permeation testing, the tissues were placed into 3D-printed permeation devices (Veit et al., 2022a), transepithelial electrical resistance was measured to confirm tissue integrity using a Millicell ERS-2 voltohmmeter (Millipore, MERS00002), then they were placed into a 12-well plate containing DPBS (0.75 mL/well, receiver solution). Treatments (0.1 mL in DPBS) were placed onto the apical side of the tissue and the tissues were placed in a humidified incubator (5% CO_2_, 37°C) until collection. HA-BODIPY was used at 6.67 mg/mL, C-HA and G-HA were treated at 20 mg/mL, cysteine (C) and reduced glutathione (G) were treated at the equivalent concentration of the respective C-HA and G-HA batches based on conjugation efficiency. At indicated timepoints, receiver solutions were collected and analyzed. HA-BODIPY concentrations were determined via fluorescence measurements as outlined above (Section 2.8). C-HA, C, G-HA and G concentrations were quantified by thiol detection as outlined in Section 2.5. SEC-MALS was used to confirm that the drug cargo did not detach during tissue permeation. MTT assay was used to determine tissue cytocompatibility as previously described (Veit et al., 2023). Briefly, treatment (100 µL) was placed on the apical side of the tissues and incubated for 24 h. The treatments were then gently washed off with DPBS and placed into a 24-well plate prefilled with MTT in media (300 μL, 1 mg/mL per well). The tissues were incubated for 3 h, washed with DPBS, then immersed in isopropanol (2 mL per tissue) with shaking for 2 h to extract the formazan dye. The extract absorbance was read at 570 nm and all samples were normalized to vehicle controls.

### 2.10 Cellular cytocompatibility

HEI-OC1 cells were seeded in a 96-well plate at 1.5 × 10^4^ cells/well in 100 µL of DMEM media with 10% FBS and incubated at 33°C/10% CO_2_ overnight. Subsequently the media were aspirated, replaced with sterile filtered media containing HA conjugate and controls, respectively, and plates were incubated overnight. For the LDH assay, 45 min prior to end of treatment, lysis buffer (10 µL/well) was added to the lysis control wells and sterile water (10 µL/well) was added to all other wells. The plate was returned to incubator for 45 min, then supernatant (50 µL) was transferred to a separate 96-well plate and mixed with LDH reagent (50 µL). This was incubated for 30 min at RT before stop solution (50 µL) was added, mixed, and absorbance was read in a plate reader (490 nm, ref. 680 nm). Absorbance was reference and blank (no cell wells) corrected, then normalized to lysis control (=100%). For the MTS viability assay, treatments were removed and replaced with MTS reagent (20 µL/well) and media (100 µL/well), then returned to incubator for 1.5 h. Absorbance was then read at 450 nm with a plate reader, blank (no cell wells) corrected, then normalized to untreated controls (100% viability).

### 2.11 Acellular superoxide scavenging assay

Initially, reagent stocks were prepared: NADH (1 mM in 0.01 M Tris-HCl buffer, pH8.5), NBT (1 mM in water), PMS (1 mM in water), which were then diluted to the final assay concentrations (with 16 mM Tris-HCl buffer, pH 8.0). NADH (500 μM, 50 μL), NBT (300 μM, 50 μL), and vehicle control (DPBS, 100 µL) or HA-drug conjugate (2 mg/mL in DPBS, 100 µL) were each added to a 96-well plate and mixed. PMS (25 μM, 50 µL) was then added to initiate the reaction, mixed, and incubated for 10 min at room temperature. Sample absorbance was then read at 560 nm (reflective of superoxide-mediated formazan production). Blank wells had PMS replaced with 50 µL of nanopure water. Ascorbic acid (1.76 mg/mL in DPBS) in place of HA-drug conjugate was used as a positive control (Mandal et al., 2011; Park et al., 2021).

### 2.12 Acellular hydrogen peroxide scavenging assay

HA-drug conjugates (20 μL, 2 mg/mL in DPBS) or control (20 μL, DPBS) were loaded into a UV-transparent 96-well plates. Hydrogen peroxide (180 μL, 40 mM in water) was added to the sample wells, 180 µL of PBS was added to the blank wells. Absorbance at 230 nm (reflective of the amount of hydrogen peroxide present) was measured every 30 min for 3 h, only 3 h data is presented (Ali et al., 2020).

### 2.13 Cellular superoxide anion assay

A Millipore Sigma Superoxide Anion Assay Kit was used for this assay with slight modifications from manufacturer’s protocol. Menadione stock (1 mM) was prepared in nanopure water + 10% DMSO, a 100 µM working solution was then prepared by diluting menadione stock in assay buffer. The following respective volumes (in µL) of each kit component (assay buffer, luminol, enhancer, SOD, conjugate (15 mg/mL in DPBS), DPBS) were added to respective wells of an opaque 96-well plate and mixed: Control—68, 5, 5, 0, 0, 20; Menadione—68, 5, 5, 0, 0, 20; SOD + Menadione - 67, 5, 5, 1, 0, 20; Conjugate + Menadione—68, 5, 5, 0, 20, 0. HEI-OC1 cells (4×10^5^ cells/well, 100 µL/well) was then added to each well and mixed. Menadione working solution (100 μM, 2 µL) was quickly added to all wells except the control which received the vehicle (1% DMSO in assay buffer, 2 µL). The plate was immediately placed in plate reader, shaken for 10 s, then luminescence was read every 3 min for 30 min.

### 2.14 Cellular peroxide assay

HEI-OC1 cells were plated in a 96-well plate (8 × 10^3^ cells/well, 100 µL/well) in DMEM media + 10% FBS and allowed to adhere overnight. The next day CM-H_2_DCFDA (1 mM stock solution) was diluted with cell culture media to a final concentration of 1.5 µM. The dye (100 µL) was then added to all wells except the no dye control and incubated for 45 min. The dye was then aspirated and replaced with 100 μL of treatment and stressor in HBSS + 2% FBS. The stressor and/or treatment were then incubated for 45 min. After incubation the fluorescent signal was read with the plate reader. The wells were then aspirated and washed with 150 μL of HBSS + 2% FBS. Fresh HBSS + 2% FBS (100 µL) was added to all wells and the fluorescent signal was read a second time.

### 2.15 Statistics

Normality testing and statistics were performed using the tests outlined in each respective figure caption using Prism version 10 (GraphPad, San Diego, CA, United States). Statistical significance was defined as a *p*-value of <0.05.

## 3 Results

### 3.1 Evaluation of HA MW effects on tissue permeation and cellular internalization

**FIGURE 1:**
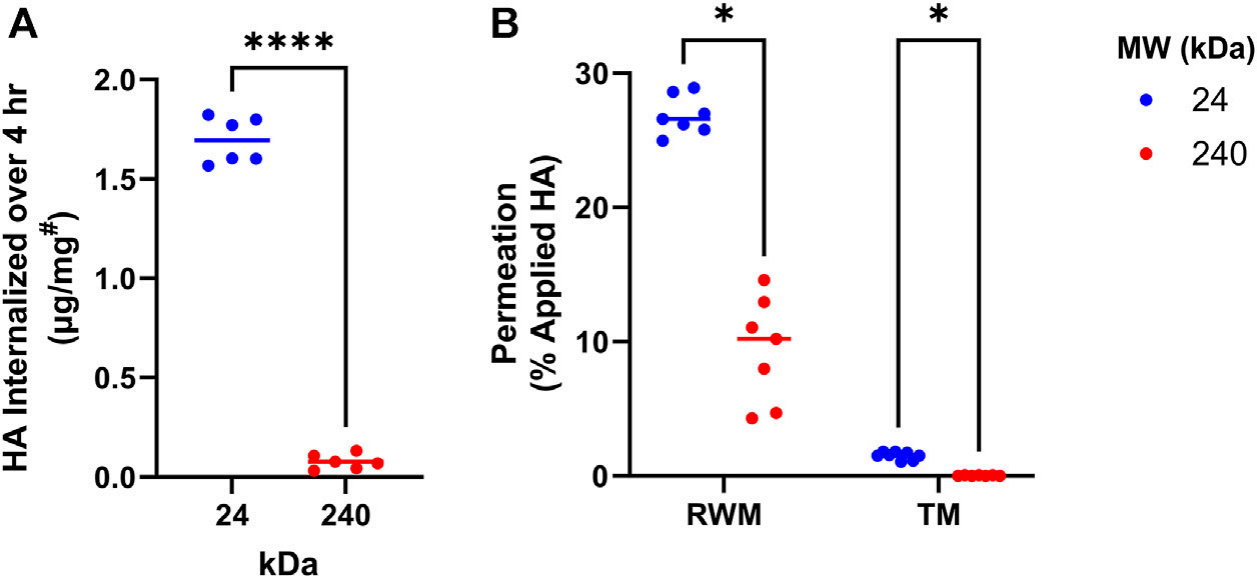
HA MW effects on cellular internalization and membrane permeation. **(A)** Internalization of 24 or 240 kDa HA-BODIPY in HEI-OC1 cells over 4 h. Cells were grown to confluence in 6-well plates and treated with 250 μg/mL HA-BODIPY. Results were normalized using a BCA assay to correct for technical variability. ^*#*^ *µg of HA-BODIPY per mg of total protein lysate*. *n* = 6; Welch’s *t*-test. **(B)** Percent of 24 or 240 kDa HA-BODIPY which permeated across TM and RWM permeation models over 24 h. TM and RWM models were placed in permeation devices and HA-BODIPY (0.1 mL of 6.7 mg/mL) was placed on apical surface. After 24 h, the basal solution was collected and permeated amount was determined. *n* = 7; multiple Welch’s T-tests with Holm-Šídák correction. **(A, B)** *p < 0.05, ****p < 0.0001, ns, not significant. HA, hyaluronan; RWM, round window membrane; TM, tympanic membrane; MW, molecular weight.

As the first step in the development of HA-antioxidant conjugates, we sought to understand the effects of HA MW on tissue permeability and cellular internalization. For this, fluorescently labelled HA derivatives (HA-BODIPY) of 2 MWs (24 kDa and 240 kDa) were tested *in vitro* for cellular internalization by using mouse cochlear cells (HEI-OC1) (Figure 1A). Our results indicate that over 20-times more 24 kDa HA-BODIPY was internalized in HEI-OC1 cells compared to the 240 kDa counterpart. We next sought to understand the internalization kinetics of the 24 kDa HA-BODIPY. Our data indicate that the internalization process was rapid, with 45% of the total amount detected internalized within 15 min, and the maximum amount internalized by 2 h (Supplementary Figure S1).

Next, the tissue permeation capability of the two HA-BODIPY molecules was investigated in *in vitro* TM and RWM tissue permeation models. Similar to our cellular data, the permeation experiments indicated a clear interdependence between permeation efficiency and MW, with the lower MW molecule showing significantly higher permeation than its higher MW counterpart (Figure 1B).

### 3.2 Synthesis of HA-Antioxidant conjugates

With an understanding of the effects of MW on tissue permeation and cellular internalization, we next sought to understand the synthesis parameters that would yield conjugates of the desired MW. Therefore, we investigated the correlation between the MW of the starting HA and the MW of the final conjugate. For this, five different MW of HA (31, 146, 285, 608, and 1,030 kDa) were employed for intermediate CMHA syntheses. The high viscosity of the 1,030 kDa HA sample presented a significant challenge to solubilization in the required reaction conditions, and was therefore excluded from subsequent evaluations. The structures of the four different MW CMHA intermediates were confirmed by ^1^H-NMR (Supplementary Figure S2), and the obtained conjugation efficiencies were determined to be 51%–57% (additional -COOH moieties). Moreover, we determined the MW of all the obtained intermediates (Supplementary Table S1). All obtained CMHA intermediates were readily water soluble, and therefore suitable for subsequent conjugation to primary amine-containing small molecules (i.e., antioxidant drugs) using previously outlined carbodiimide chemistry (Arrigali and Serban, 2022).

**FIGURE 2:**
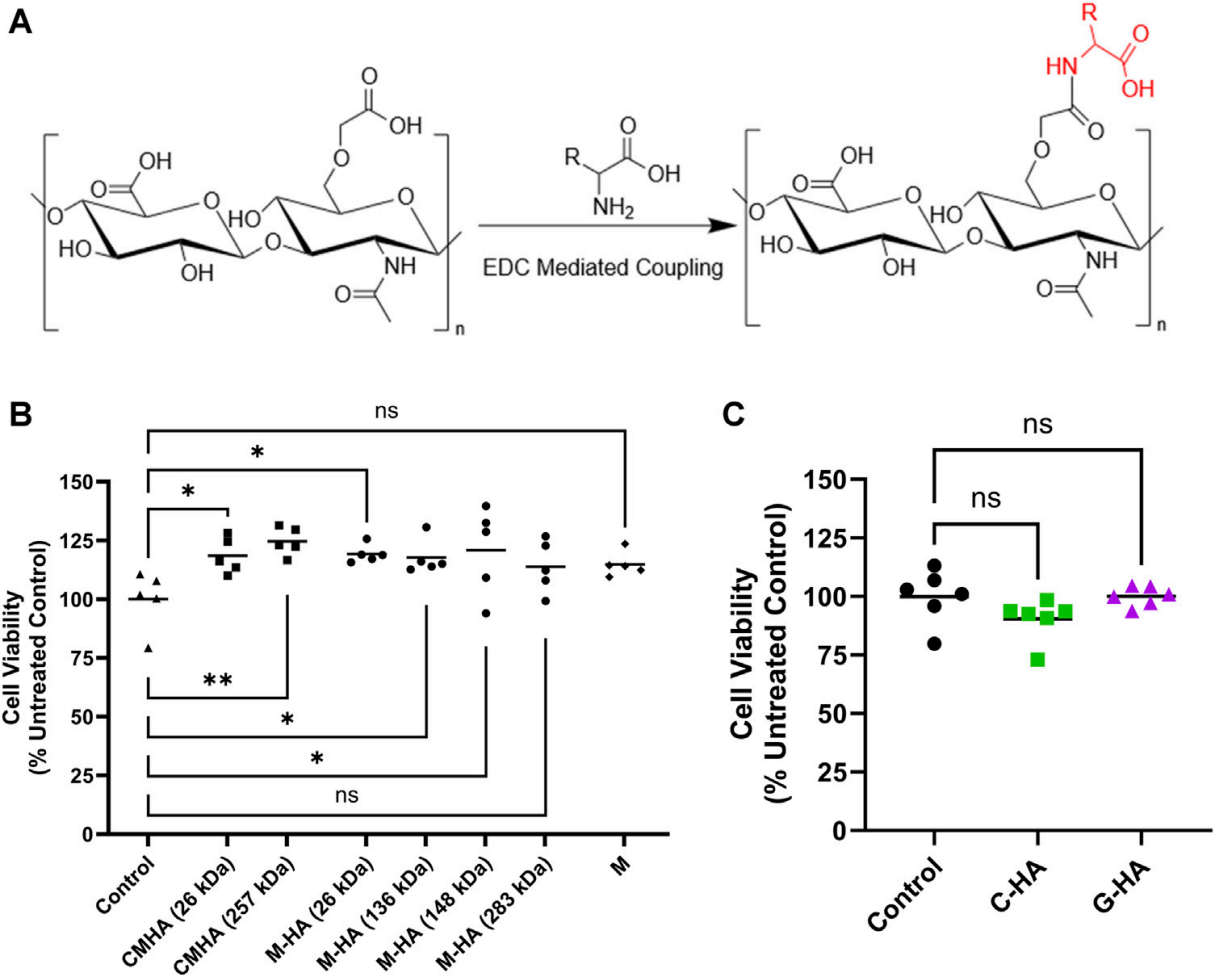
Reaction scheme and cytocompatibility of HA-antioxidant conjugates. **(A)** Reaction scheme used for synthesis of HA-antioxidant conjugates. *R, rest of the molecule.* **(B)** MTS cytocompatibility assay of various molecular weights of 1.5 mg/mL CMHA or M-HA applied to HEI-OC1 cells for 24 h *n* = 5 **(C)** MTS cytocompatibility assay of 1.5 mg/mL C-HA and G-HA in HEI-OC1 cells treated for 24 h *n* = 6. **(B and C)** One-way ANOVA with Dunnett’s correction; *p < 0.05, **p < 0.01; ns, not significant. C, cysteine; CMHA, carboxy methyl HA; G, reduced glutathione; HA, hyaluronan; M, D-methionine.

Building on our previous work (Arrigali and Serban, 2022), M-HA was then synthesized with the four different MW CMHAs and conjugate structures were confirmed via ^1^H-NMR (Supplementary Figure S3). A general reaction scheme for the syntheses of all conjugates is presented in Figure 2A. The four resulting different MW M-HAs were then tested in HEI-OC1 cells to assess the potential impact of different MW on cytocompatibility (Figure 2B). No statistically significant differences in cell viability were observed between the conjugates tested, indicating that the MW of the antioxidant carrier is not affecting cellular compatibility. Based on these results, the 31 kDa HA starting material was selected for all subsequent conjugates.

M-HA, C-HA, and G-HA conjugates were water-soluble, and the successful covalent conjugation of the antioxidants was confirmed by ^1^H-NMR (Supplementary Figure S4). The obtained conjugates were extensively characterized for MW, conjugation efficiency, polydispersity index (Đ), refractive index increment and zeta (ζ) potential (Supplementary Table S2). The M-HA conjugate had a weight-average MW (M_w_) ranging from 26.0 to 26.4 kDa, a Đ of 1.262–1.382, and a conjugation efficiency of 7.85%–7.90% w/w (antioxidant mass to total conjugate mass). The C-HA conjugate had a M_w_ ranging from 31.9 to 32.0 kDa, a Đ of 1.230–1.349, and a conjugation efficiency of 5.96%–5.97% w/w. G-HA had a M_w_ ranging from 31.5 to 34.4 kDa, a Đ of 1.287, and a conjugation efficiency of 22.26%–26.12% w/w. C-HA and G-HA were also assessed in HEI-OC1 cells, with our data indicating adequate cytocompatibility for both conjugates (Figure 2C; Supplementary Figure S5).

### 3.3 Acellular oxidative protection screenings

**FIGURE 3:**
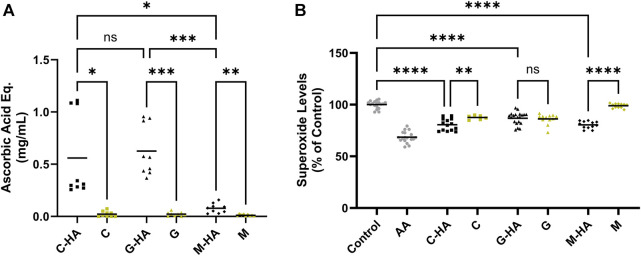
Acellular ROS-scavenging assays. **(A)** Acellular peroxide scavenging assay presented in ascorbic acid equivalence units. Each conjugate was tested at 1.5 mg/mL and compared to its respective unconjugated antioxidant at the equivalent concentration determined by its conjugation efficiency. Higher values indicate improved peroxide scavenging. *n* = 9 **(B)** Acellular superoxide assay. Each conjugate was tested at 2 mg/mL, its respective unconjugated antioxidant was tested at the equivalent concentration. AA (1.76 mg/mL) was included as a positive control. Lower values indicate improved superoxide scavenging. *n* = 12–20. **(A, B)** Brown-Forsythe and Welch one-way ANOVA with Dunnett’s T3 correction; *p < 0.05, **p < 0.01, ***p < 0.001, ****p < 0.0001, ns, not significant. AA, ascorbic acid; C, cysteine; G, reduced glutathione; HA, hyaluronan; M, D-methionine.

We next sought to assess the effect of conjugation on the antioxidant activity of M, C, and G. For this, we employed acellular assays to unequivocally, without any background interference, assess the conjugates’ inherent antioxidant activity. Our data indicate that all three conjugates reduced peroxide radical levels (Figure 3A, reported in ascorbic acid equivalence units) with C-HA and G-HA showing statistically higher protective efficiencies than M-HA. Additionally, all three conjugates performed significantly better than their respective unconjugated antioxidants. In the superoxide radical scavenging assay, all three conjugates were comparably effective at reducing superoxide levels (Figure 3B). C-HA and M-HA were able to scavenge superoxide species better than their respective unconjugated drugs, whereas G-HA scavenged at a level equivalent to unconjugated G. Based on their performance in these assays, which indicate higher overall scavenging potential, C-HA and G-HA were selected as lead compounds for further evaluation.

### 3.4 Cellular oxidative protection screening

**FIGURE 4:**
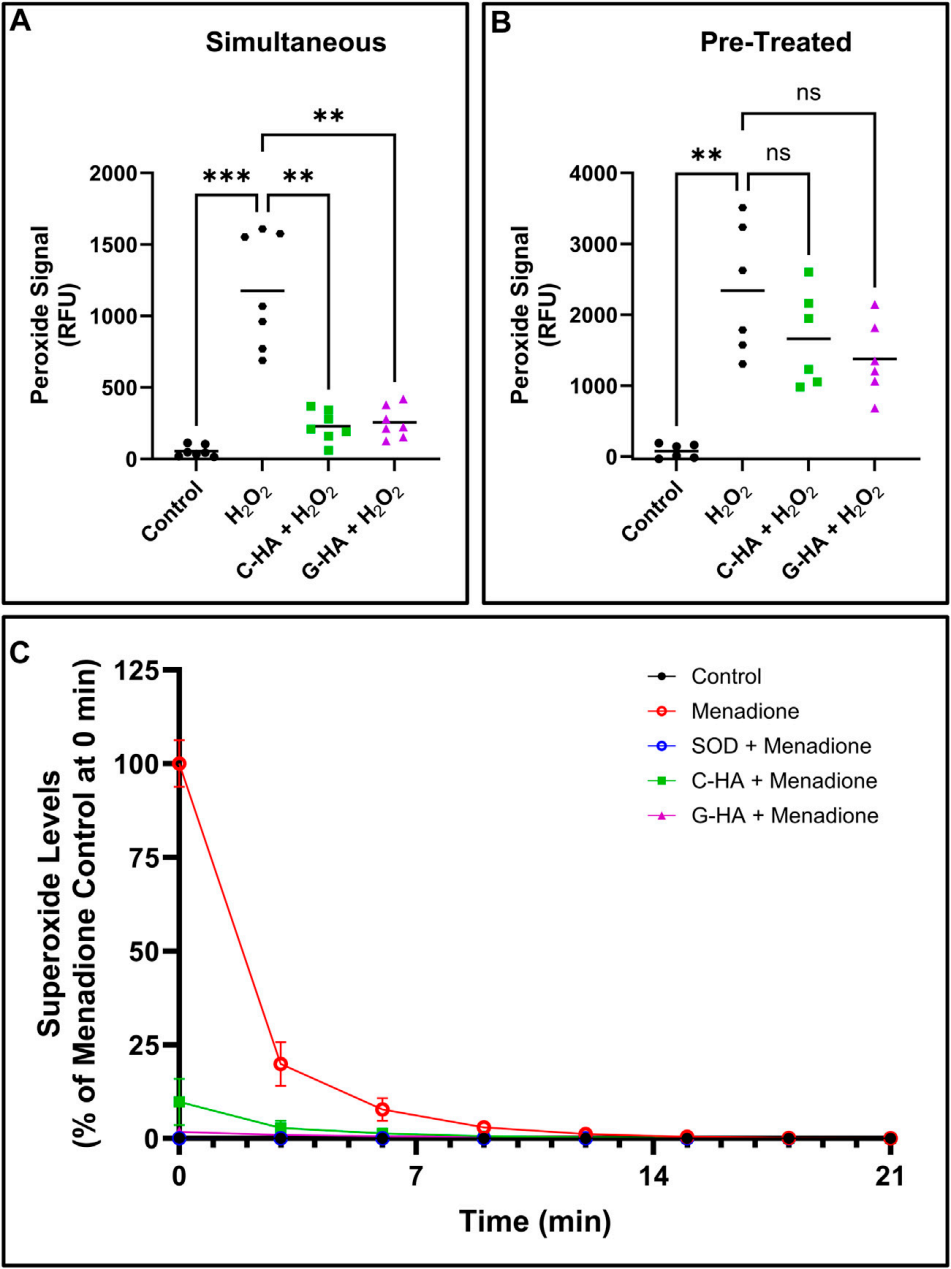
Cellular ROS-scavenging assays. Cellular peroxide scavenging assays performed by treating HEI-OC1 cells stressed by 1 mM hydrogen peroxide (H_2_O_2_) with 1.5 mg/mL of C-HA or G-HA. Conjugates were either treated **(A)** simultaneously with the stressor, or **(B)** as a 24 h pre-treatment. *n* = 7; Brown-Forsythe and Welch ANOVA with Dunnett’s T3 correction; **p < 0.01, ***p < 0.001, ns, not significant. **(C)** Cellular superoxide sequestering kinetics in HEI-OC1 cells which were induced to produce superoxides with 10 µM menadione and treated with 1.5 mg/mL C-HA or G-HA. SOD was used as a control to verify the observed response from menadione was due to superoxide formation. n = 8. Graph shows mean ± SD. C, cysteine; G, reduced glutathione; HA, hyaluronan; M, D-methionine, SOD, superoxide dismutase.

The two lead conjugates were then tested for antioxidant activity in cell-based assays. First, conjugates and stressor (1 mM H_2_O_2_) were simultaneously added to cells for 45 min, then the total peroxide level was measured (Figure 4A). Both conjugates significantly reduced peroxide levels relative to the stressed control. Next, cells were first pre-treated with conjugates for 24 h, then challenged with stressor for 45 min (Figure 4B). Unlike in the simultaneous treatment, pre-treatment appeared to have marginal protective effects, which were not statistically significant compared to the stressed control. A superoxide assay was also performed, in which conjugates were applied to cells in conjunction with menadione, which was used to induce cellular superoxide generation (Figure 4C). In this assay, both C-HA and G-HA drastically and rapidly reduced cellular superoxide levels, with G-HA showing an immediate reduction to nearly baseline levels.

### 3.5 Conjugates *in vitro* tissue permeation

**FIGURE 5:**
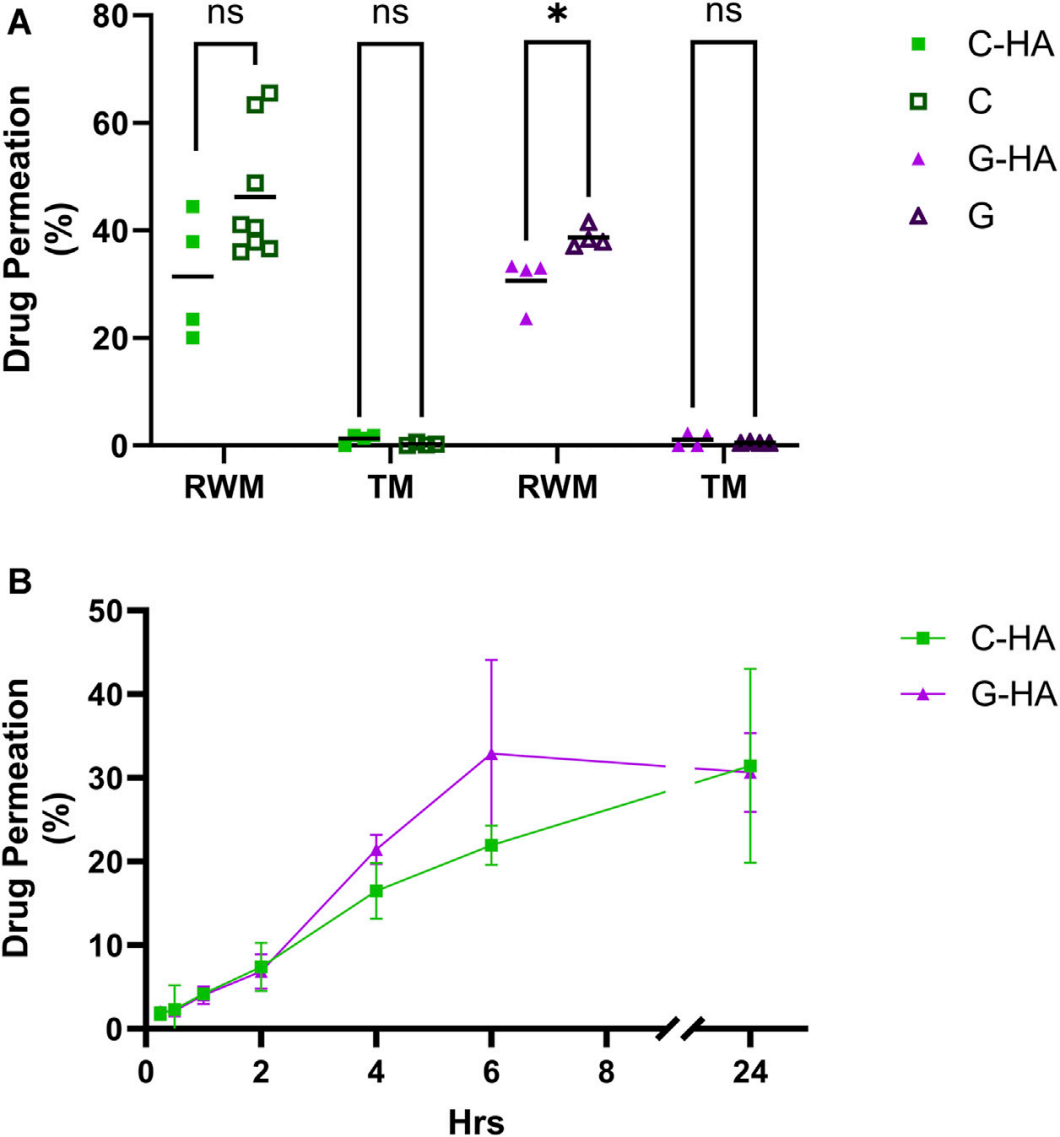
Conjugate permeation experiments. **(A)** Permeation of 0.1 mL of 20 mg/mL C-HA or G-HA and the equivalent concentration of their respective unconjugated antioxidants across the *in vitro* TM and RWM permeation models over a 24 h period. *n* = 4–8; independent *t*-tests; *p < 0.05, ns, not significant. **(B)** Permeation kinetics of C-HA and G-HA across the RWM model. *n* = 3–4. Graph shows mean ± SD. C, cysteine; G, reduced glutathione; HA, hyaluronan; RWM, round window membrane; TM, tympanic membrane.

To test the conjugates’ potential as topical therapeutics, we assessed their permeability across physiologically-representative *in vitro* TM and RWM permeation models previously developed by our group (Veit et al., 2022a; Singh et al., 2022). The conjugates, as well as the corresponding equivalent amounts of unconjugated antioxidants, were placed on top of the tissues and the amount of each permeated at various time points was quantified (Figures 5A, B). For TM, there were no significant differences in the permeated amounts of conjugates *versus* unconjugated drugs, and overall the permeated amount at 24 h was just above 1% of total compound applied. Our data also indicate that C-HA and G-HA can readily permeate the RWM model, with 16% and 21%, respectively, of the total applied drug permeating within 4 h and over 30% permeating within 24 h. However, in the RWM there was a slight, but statistically significant, difference in G-HA permeation compared to G alone, while no difference was observed between C-HA and C. Finally, we also assessed the viability of the RWM and TM models after a 24 h exposure to the conjugates (Supplementary Figure S6A). As seen in the cochlear cell cytocompatibility assays, both treatments were well tolerated in the tissue models, with no cytotoxic effects.

## 4 Discussion

This study aimed to develop a possible therapeutic or prophylactic against NIHL by targeting the associated oxidative stress in the cochlea (Kamata et al., 2005; Dinh et al., 2015a; Kurabi et al., 2017a; Frye et al., 2019). Our investigation of the permeability and internalization of two different MW of fluorescently labeled HAs, revealed that a lower MW appears to more readily pass through both cellular membranes and tissue barriers. This result is intuitive and agrees with classical expectations of diffusion and permeation dynamics favoring smaller MW molecules over larger ones. These results informed our decision to synthesize subsequent HA-antioxidant conjugates with a lower MW HA.

For conjugation, we selected compounds with sulfur containing moieties (thioether in methionine, thiol in cysteine and glutathione) due to their well-documented antioxidant properties, as well as the availability of a structural primary amine that makes them suitable for convenient covalent attachment via a carbodiimide reaction. Additionally, these three antioxidants have been previously studied in the context of reducing ROS-mediated hearing loss and have shown promising protective effects (Kamata et al., 2005; Campbell et al., 2007; Pecha et al., 2020; Campbell et al., 2021).

Our conjugate synthesis scheme involves the generation of a CMHA reaction intermediate prior to conjugation of the antioxidant. This intermediate adds additional carboxyl moieties to HA to allow for more efficient conjugation with the primary amine of the antioxidants. Since each reaction step occurs at a different pH, the MW of the initial HA starting material will decrease due to depolymerization. Our data indicate that the use of 31 kDa HA was optimal for generation of conjugates with MWs shown to be suitable for maximum tissue and cellular membrane permeation.

Building on our previous work, we then synthesized HA conjugates with M by using HA starting materials of different MWs. The resulting conjugates were tested in cochlear cells and our results indicated no size-dependent cytocompatibility issues. Taken together with our previous findings, these data informed the subsequent syntheses of C-HA and G-HA conjugates based only on the 31 kDa HA starting material. These conjugates were also shown to be cytocompatible with cochlear cells.

Acoustic trauma is associated with oxidative stress and increased ROS generation, and studies have shown that boosting antioxidant defense attenuates NIHL (Campbell et al., 2007; Mao and Chen, 2021). Mechanistically, acoustic trauma leads to ROS release by the marginal cells of the stria vascularis, which induces subsequent intrinsic and extrinsic apoptotic signaling in hair cells (Dinh et al., 2015a). Peroxide generation has been associated with NIHL (Le Prell et al., 2019), and *in vivo* studies have indicated acoustic trauma results in the presence of superoxides in the cochlea (Yamane et al., 1995). Consequently, we employed two different acellular assays to assess, without any cell-specific background interference, the inherent antioxidant capabilities of the conjugates. The first peroxide assay reports the data in the commonly used ascorbic acid equivalence unit, which is defined as the amount of ascorbic acid needed to achieve the same antioxidant activity as the test compound. Our data indicates that C-HA, G-HA, and M-HA were all able to scavenge peroxides significantly better than their respective unconjugated antioxidants, with C-HA and G-HA outperforming M-HA. For compounds such as C or G that contain free thiols, hydrogen peroxide neutralization to water occurs via their oxidation and subsequent formation of disulfide bonds (forming cystine and glutathione-oxidized, respectively) (Combs and DeNicola, 2019). This process depends on the physical proximity of two molecules, and therefore, we hypothesize that the observed enhanced antioxidant effects of conjugates are due to the concentrated localization of the antioxidants attached to HA polymeric chains, compared to the free floating unconjugated drugs. The second acellular assay evaluated the compounds’ efficiency against superoxides. All three conjugates showed statistically significant reductions in superoxide levels compared to the controls. Cumulatively, the two acellular assays indicate that the conjugates protect against both superoxides and peroxides. Based on the peroxide data, and with the intent to advance the most effective conjugates, C-HA and G-HA were selected as lead candidates for further testing in cellular assays.

We next assessed the ability of our lead conjugates to scavenge ROS in cellular assays. A peroxide assay was tested in two conditions, one involving the simultaneous application of conjugate and peroxide, and the other which pre-treated the cells with conjugate prior to the peroxide stressor. With NIHL, ROS are rapidly produced in the inner ear and can diffuse via the endolymph to the rest of the inner ear cells (Nicotera et al., 2003; Dinh et al., 2015a; Raju, 2015; Kurabi et al., 2017a). Therefore, an assay assessing the conjugate’s ability to scavenge ROS prior to entering the cells is physiologically relevant, and we postulate that if the conjugates could target the peroxide species extracellularly, they would be able to protect the cells from damage. These ROS peroxide assays use a fluorescent dye, which is activated by ROS and emits a fluorescent signal proportional to the amount of peroxide present. Simultaneous treatment, but not pre-treatment, with C-HA and G-HA showed a reduction in peroxide signal. One possible reason for the lack of efficacy with pre-treatment may be due to treatment timing (24 h prior to stressor), which may need to be reduced to observe a prophylactic effect. Future studies could explore variable treatment times and their effects on antioxidant activity and protection. The superoxide assay used simultaneous treatment of conjugates with menadione, an intracellular superoxide-generator (Fukui et al., 2012). Our data indicated that C-HA and G-HA treated stressed cells had significantly less superoxide anion levels and a more rapid return to baseline than the stressed-alone cells. These cellular results are in alignment with the previous acellular data and further reinforce the therapeutic potential of C-HA or G-HA against ROS-mediated NIHL.

Since the damage from NIHL primarily occurs in the cochlea, our expectation is that a topical therapeutic would need to, at a minimum, permeate the RWM, and ideally, both TM and RWM. To test this, we used *in vitro* permeation models of the TM and RWM previously developed by our lab for drug permeability testing (Veit et al., 2022a; Veit et al., 2022b; Singh et al., 2022). Both C-HA and G-HA were able to permeate the TM model, but at a significantly lower percentage than the RWM. This is expected as the TM consists of a skin-like epidermal outer layer composed of a difficult to permeate corneal layer and containing abundant intercellular tight junctions (Yang et al., 2017). Conversely, the RWM is composed of epithelial cells which form weaker tight junctions and do not contain a corneal layer, therefore, it is expected to more easily facilitate paracellular diffusion. Previous studies show that the TM is significantly more difficult to permeate than the RWM and therefore, would be the limiting factor for a solely topical approach (Veit et al., 2022b). If further studies find that the amount of conjugate permeating the TM is inadequate for the desired therapeutic effect, options to bypass the TM such as intratympanic injections, can be explored. Our results did not show an increase in permeability of the conjugate compared to an equivalent amount of unconjugated antioxidant. However, it is important to note that minimal differences were observed between the tissue permeation of the large conjugated antioxidants (C-HA = 32–38 kDa, G-HA = 32–34 kDa) and the antioxidants alone (cysteine = 121 Da, glutathione = 307 Da) despite the significant MW/size differences. This suggests that HA conjugation remains a valid approach to the development topical treatments.

Overall, this study presents the successful synthesis and characterization of novel HA-antioxidant conjugates that show promise as potential therapeutics against oxidative stress-induced NIHL. Specifically, we showed there is a MW-dependent effect on HA cellular internalization and tissue permeation, which informed the selection of lower MW HA starting materials for the syntheses of HA-antioxidant conjugates. Moreover, we demonstrated that the conjugates were cytocompatible and showed adequate antioxidant activity in two acellular assays. The outcomes of these assays informed the selection of the two lead compounds, C-HA and G-HA, that were then shown to be effective antioxidants in cell-based assays. Additionally, we showed that both lead conjugates were able to effectively permeate the RWM. Since the lead conjugates showed minimal TM permeation, future studies would need to be performed to assess whether this permeation is sufficient to achieve the desired therapeutic effect. If TM permeation is not sufficient, alternative permeation enhancers such as intratympanic injections, microneedle devices, or chemical penetrants could be explored. While further *in vivo* studies are needed to confirm these findings, our data supports the possibility of exploring these conjugates as topical, minimally-invasive therapeutics for NIHL.

## Data Availability

The raw data supporting the conclusion of this article will be made available by the authors, without undue reservation.
