# Supplementary material for: In vitro characterization of novel hyaluronan-antioxidant conjugates as potential topical therapeutics against hearing loss
Source: Front Pharmacol. 2024 Feb 28;15:1355279. doi: 10.3389/fphar.2024.1355279 (PMC10933124; doi:10.3389/fphar.2024.1355279)
Supplement: Supplementary file 1 [file DataSheet1.pdf]

Supplementary Materials

In Vitro Characterization of Novel Hyaluronan-Antioxidant Conjugates as Potential Topical Therapeutics against Hearing Loss

Elizabeth M. Arrigali[1†], Joachim G.S. Veit[1,2†], Bhaskar Birru[1†], Jack Van Tine[1], Kolton C. Sandau[1], Emma Barrett-Catton[1], Zachary Tonnerre[1], *Monica A. Serban[1,2*]

[†]These authors contributed equally to this work and share first authorship

[1]Department of Biomedical and Pharmaceutical Sciences, University of Montana, Missoula, MT

[2]Montana Biotechnology Center (BIOTECH), University of Montana, Missoula, MT

***Correspondence:**

Monica Serban

monica.serban@umontana.edu

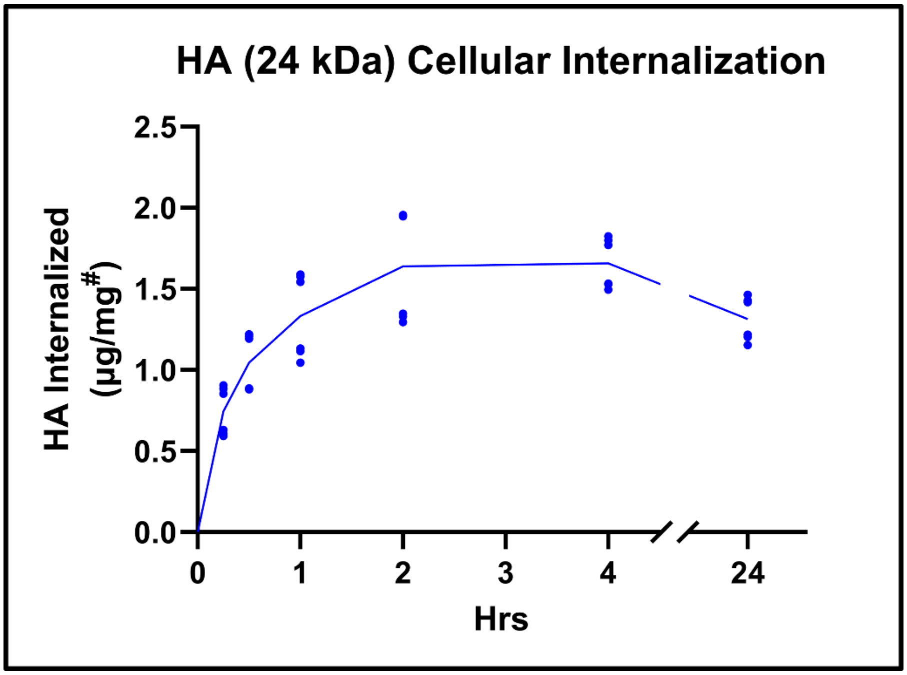

Supplemental Figure S1: Internalization kinetics of 24 kDa HA-BODIPY. Internalization kinetics of 24 kDa HA-BODIPY in HEI-OC1 cells over 24 hr period. n = 4. [#]*µg of HA-BODIPY per mg of total protein lysate. HA, hyaluronan.*

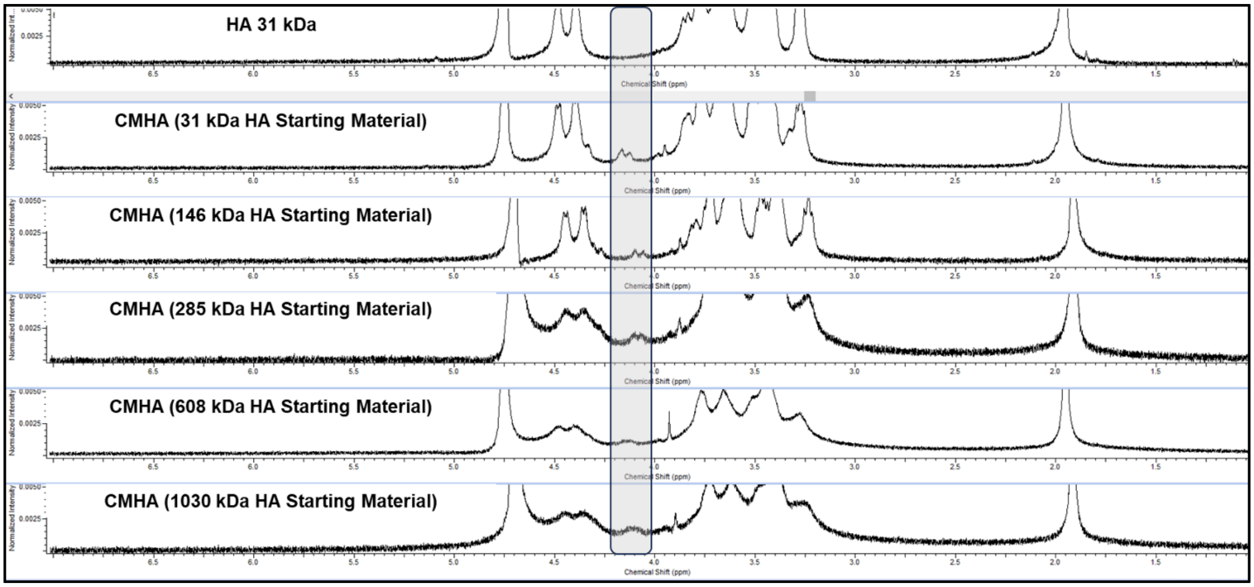

Supplemental Figure S2: ^{1}H-NMR spectra of different molecular weight CMHA. Molecular weight of starting materials from low to high going down in the figure. All NMRs performed in deuterated water. The top spectrum is the HA starting material and the bottom five spectra are after the carboxymethyl addition. The shaded box highlights the peaks from the methylene protons of the carboxymethyl moiety. *CMHA, carboxymethyl-HA; HA, hyaluronan.*

Supplemental Table S1

HA Starting Material			Resulting CMHA		
M_w (kDa)	PDI (Đ)	dn/dc (mL/g)	M_w (kDa)	PDI (Đ)	dn/dc (mL/g)
31.1 ± 2.6	1.322 ± 0.060	0.1435 ± 0.0013	25.6 ± 1.3	1.372 ± 0.005	0.1463 ± 0.0027
146.4 ± 2.6	1.413 ± 0.025	0.1502 ± 0.0025	113.7 ± 3.9	1.344 ± 0.011	0.1441 ± 0.0015
285.0 ± 2.3	1.329 ± 0.010	0.1481 ± 0.0032	178.4 ± 2.1	1.329 ± 0.012	0.1460 ± 0.0028
607.8 ± 13.2	1.090 ± 0.011	0.1356 ± 0.0038	256.8 ± 4.4	1.365 ± 0.025	0.1460 ± 0.0016

n = 3. Values are shown mean ± SD. dn/dc, refractive index increment; M_w, weight-average molecular weight; PDI, polydispersity index.

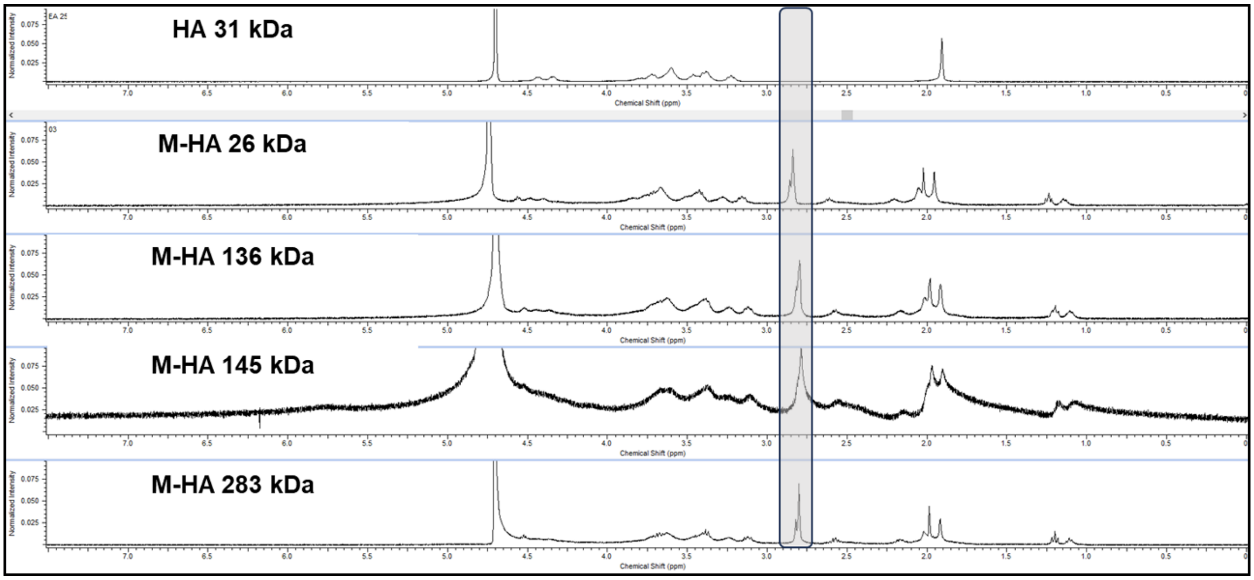

Supplemental Figure S3: ^{1}H-NMR spectra of M-HA conjugates using HA starting materials of different molecular weights in ascending order. Molecular weight listed represents the molecular weight of the HA starting material used in the initial CMHA reaction. The shaded box highlights the peak of interest that is present only after D-methionine has been conjugated to CMHA. *CMHA, carboxymethyl-HA; M-HA, methionine-HA; HA, hyaluronan.*

Supplemental Table S2

	M_w (kDa)	Conjugation Efficiency (% w/w*)	PDI (Đ)	dn/dc (mL/g)	ζ-potential (-mV)
**C-HA**	31.9-32.0	5.96-5.97	1.230-1.249	0.1446-0.1450	8.182-9.35
**G-HA**	31.5-34.4	22.26-26.12	1.287-1.287	0.1542-0.1549	12.94-15.41
**M-HA**	26.0-26.4	7.85-7.90	1.262-1.382	0.1495-0.1541	16.00-18.84

*Values are shown as min-max of replicates. *% mass of antioxidant vs total mass. dn/dc, refractive index increment; M_w, weight-average molecular weight; PDI, polydispersity index.*

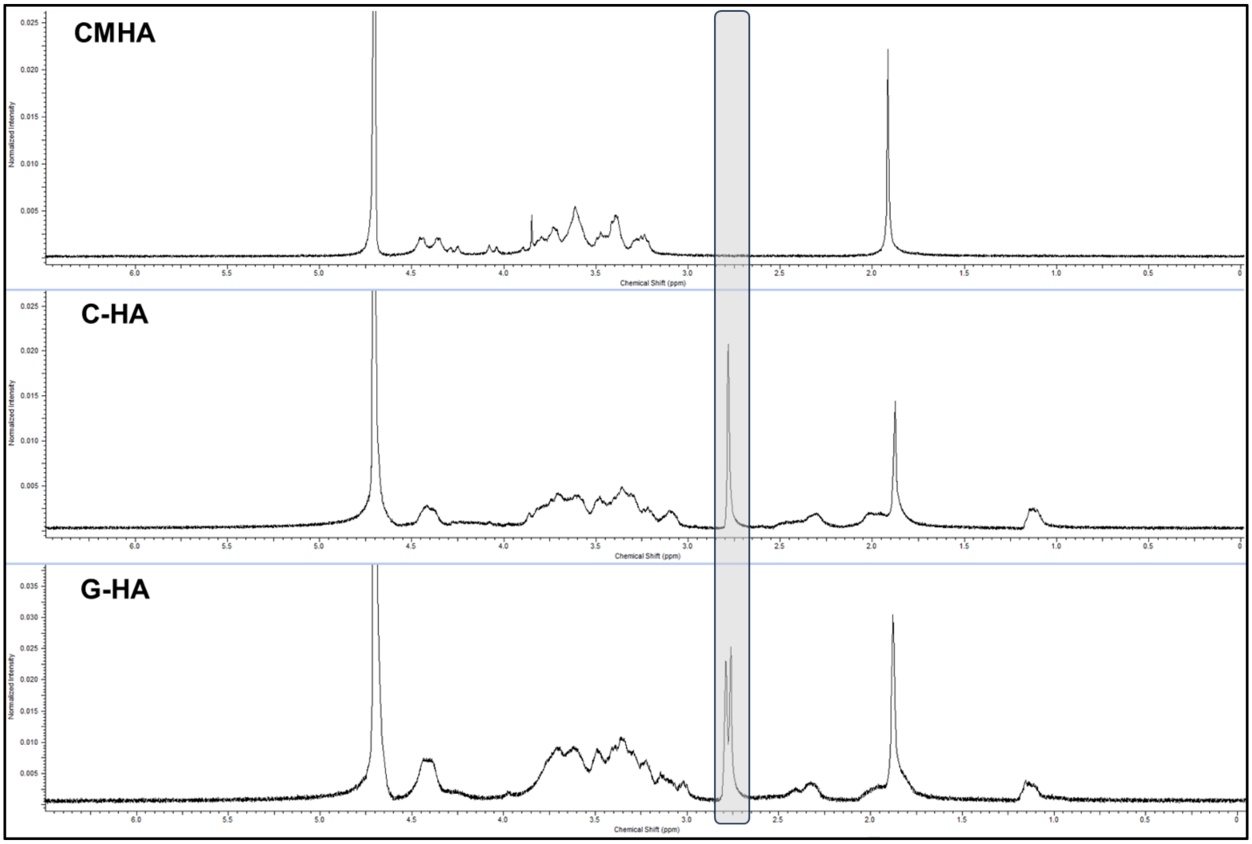

Supplemental Figure S4: ^{1}H-NMR spectra of CMHA, HA-Cysteine (C-HA), and HA-glutathione (G-HA) conjugates. NMR performed in deuterated water. The shaded box highlights the peak of interest that is present only after cysteine or glutathione have been conjugated to CMHA. *C, cysteine; CMHA, carboxymethyl-HA; G, reduced glutathione; HA, hyaluronan.*

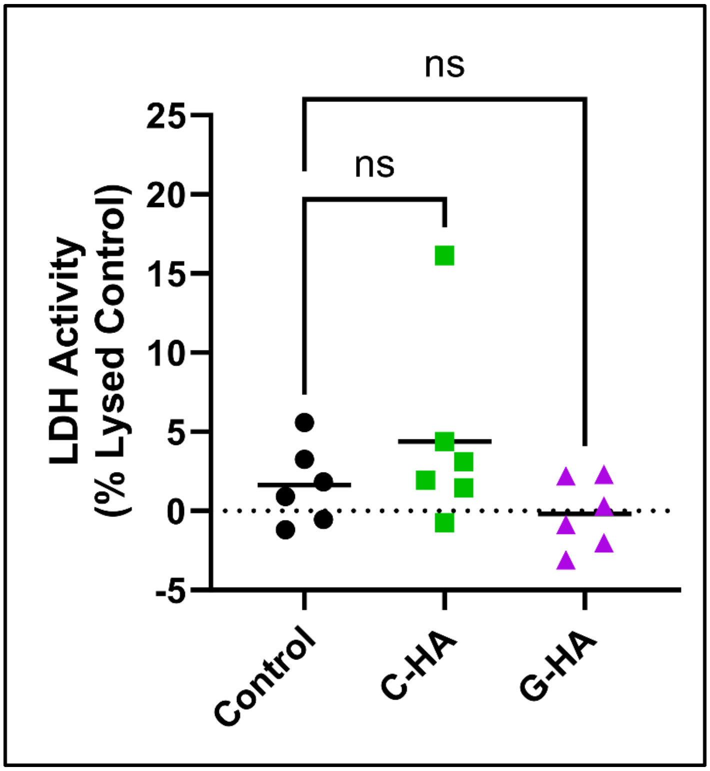

Supplemental Figure S5: LDH cytocompatibility assay of 1.5 mg/mL C-HA and G-HA in HEI-OC1 cells treated for 24 hrs. n = 6, one-way ANOVA with Dunnett's correction; ns = not significant. *C, cysteine, G, reduced glutathione; HA, hyaluronic acid; LDH, lactate dehydrogenase.*

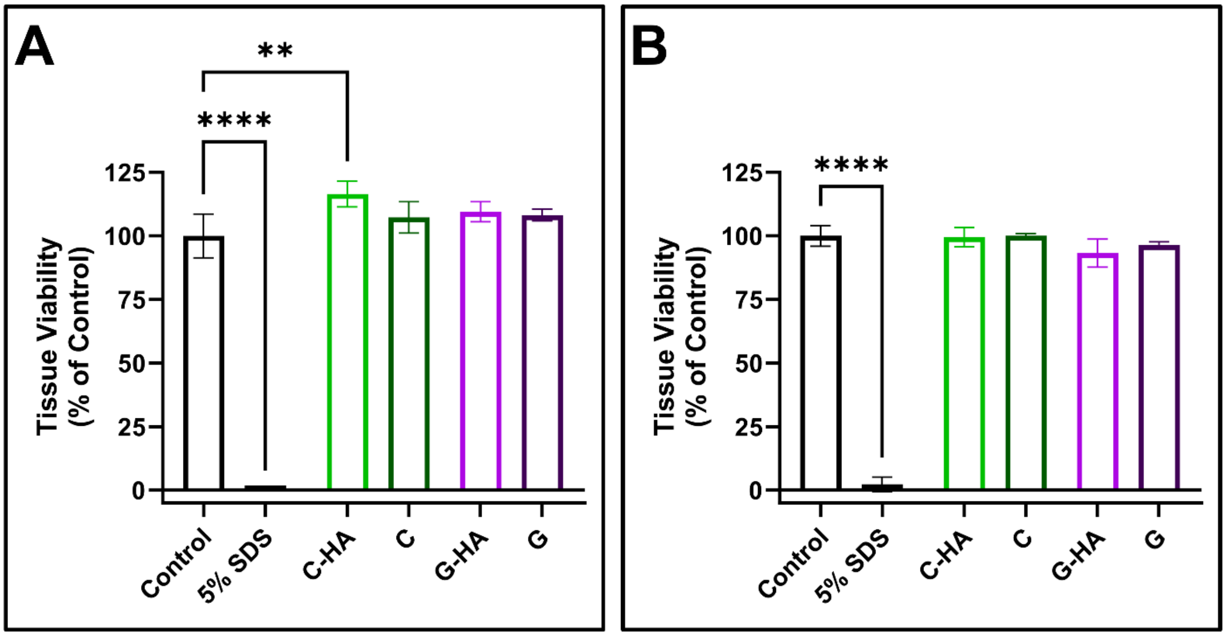

Supplemental Figure S6: Tissue viability after conjugate permeation. Tissue viability measured by MTT assay of (**A**) RWM and (**B**) TM permeation models after 24 hr exposure to 20 mg/mL of C-HA, G-HA, or the equivalent concentration of their respective unconjugated antioxidants. 5% SDS was used as a positive control for toxicity. n = 3-6; one-way ANOVA with Dunnett's correction vs control; **$p<0.01$; ****$p<0.0001$; only significant differences from control are indicated. Graphs show mean ± SD. *C, cysteine; G, reduced glutathione; HA, hyaluronan; RWM, round window membrane; TM, tympanic membrane.*
